# Drug trends among non-institutionalized Canadians and the impact of data collection changes in the Canadian Health Measures Survey 2007 to 2015

**DOI:** 10.1371/journal.pone.0214718

**Published:** 2019-04-12

**Authors:** Yi-Sheng Chao, Chao-Jung Wu, Hsing-Chien Wu, Wei-Chih Chen

**Affiliations:** 1 Centre de Recherche du Centre Hospitalier de l’Université de Montréal (CRCHUM), Université de Montréal, Montréal, Québec, Canada; 2 Département d'informatique, Université du Québec à Montréal, Montréal, Québec, Canada; 3 Taipei Hospital, Ministry of Health and Welfare, New Taipei City, Taiwan; 4 Department of Chest Medicine, Taipei Veterans General Hospital, Taipei, Taiwan; 5 Faculty of Medicine and Institute of Emergency and Critical Care Medicine, School of Medicine, National Yang-Ming University, Taipei, Taiwan; University of Brescia, ITALY

## Abstract

**Background:**

There is a global trend of increasing use in prescription and over-the-counter (OTC) drugs. This hasn’t been verified in Canada. In addition, there are changes made to the collection method of medication information after the Canadian Health Measures Survey (CHMS) cycle 2. This study aims to review the potential impact of the changes in medication data collection and the trends in medication use if data quality remains similar throughout the CHMS cycles 1 to 4. This is fundamental for the analysis of this biomonitoring database.

**Methods:**

The CHMS cycle 1 to 4 medication and household data were used to study the trends of medication use between 2007 and 2015. The use of prescription or OTC drugs was grouped based on the first levels of the Anatomical Therapeutic Chemical (ATC) Classification system. The total numbers of medications were asked in all cycles. However, only a maximum of 15 and 5 drugs could be respectively reported for existing and new prescription or OTC drugs in cycles 1 and 2. There were no restrictions on drug reporting after cycle 2. The trends of medication use were described as ratios, compared to cycle 1.

**Results:**

The total numbers of the types of medication ever identified decreased from 739 to 603 between cycles 1 and 4. The proportions of using any drugs were from 0.90 to 0.88 between cycles 1 and 4 (ratio = 1.08 in cycle 4, 95% CI = 0.89 to 1.26). The numbers of drugs in use were from 3.9 to 3.8 (ratio = 1.05 in cycle 4, 95% CI = 0.86 to 1.24). The proportions of prescription drug use were from 0.53 to 0.55 (ratio = 1.13 in cycle 4, 95% CI = 0.89 to 1.37), while the numbers of prescription were from 1.51 to 1.68 (ratio = 1.20 in cycle 4, 95% CI = 0.92 to 1.48). The use of diabetes and thyroid medication had trends similar to the respective disease prevalence. The use and the numbers of drugs for blood and blood forming organs significantly increased between cycles 1 and 4 (ratio = 1.56 in cycle 4, 95% CI = 1.03 to 2.10).

**Conclusions:**

There is an increasing trend in the use of blood and blood forming agents through cycles 2 to 4 and cardiovascular drugs in cycle 3. For diabetes and thyroid medication, the proportions of medication use increase proportionally with disease prevalence. The changes in the medication information collection method may not have important impact on the reporting of the use of prescription or OTC drugs.

## Background

There is an increasing trend of the use of prescription[1] and over-the-counter (OTC) drugs globally.[2] In the United States (US), the expenditure on prescription drugs were $456 billion in 2017, an increase of 1.7% since 2016.[3] The trend is expected to continue. The per capita expenditure on prescription drugs is expected to grow at 4% annually between 2018 and 2026.[4] The major contributors to the high growth in drug expenditure include inflation, population growth, increase in per capita prescription, and per capita prescription intensity.[5, 6] In the US, both the proportions of prescription use and per capita prescription intensity increased between 1999 and 2012.[7] Among 18 most commonly used prescription drugs, 11 of them have been used more commonly, including antihyperlipidemic agents, antidepressants, prescription proton-pump inhibitors, and muscle relaxants.[7] For specific populations, the prevalence of using prescription drugs can be as high as 81% among the Medicare enrollees in the United States.[8] In Canada, the information on the historical trends of drug use is limited. It was estimated that 41% of Canadians living in private households are currently using prescription drugs based on the estimates from the 2007 to 2011 Canadian Health Measures Survey (CHMS).[9] The most frequently used prescription medications include lipid-lowering agents, angiotensin-converting enzyme-inhibitors, peptic-ulcer drugs, and acid-reducers between 2007 and 2009 in Canada.[9] There are also other significant drug trends observed by researchers or the public, particularly the use of opioids.[10, 11] In Canada, the overall trend in medication use, such as proportions of drug use and the number of drugs used, remains unclear.

In addition, there are changes made to the data collection methods in the CHMS cycle 3, conducted between 2011 and 2013. The changes to the collection methods of the CHMS included an increase in the maximum number of prescription or OTC drugs that can be identified through the Anatomical Therapeutic Chemical (ATC) classification codes[12] or the American Hospital Formulary Service (AHFS) drug codes.[13] In cycles 1 and 2, a maximum of 15 current drugs can be identified. In cycles 3 and 4, the limits on the maximum numbers were removed.

The second change in the collection methods of the CHMS was the way the drugs were classified. In cycles 1 and 2, three categories of drugs are identified through drug codes: prescription, over-the-counter (OTC), and health products. At most 15 drug codes and five codes of new drugs can be identified for each of three categories in cycles 1 and 2. In total, at most 60 drug codes (20x3) can be documented for each CHMS interviewee in cycle 1 and 2. From cycle 3 and onwards, three drug categories were reduced to two: prescription and OTC. The restrictions on the numbers of drugs that could be reported were eliminated at the same time.

The last change is about how data are stored. In the cycle 1 and 2 data sets, each survey participant has 60 drug records in the same row. From cycle 3 and on, each drug record is stored in separate rows. This requires further data linkage for the CHMS data.

The changes to the data collection methods of the CHMS are considered challenges for the study of medication use in Canada using this data. The aforementioned analysis of the CHMS data by Rotermann et al. (2014) only used data from the CHMS cycles 1 and 2 implemented between 2007 and 2011[9] before the changes in data collection were introduced. Analysis of the CHMS medication data across four cycles and beyond require careful review of the CHMS data to account for the change in reporting.

Based on available evidence, we don’t have enough information on the drug trends in Canada and are uncertain about the impact of the changes in data collection in the CHMS. We hypothesize that there is an increasing trend of prescription drug use because increasing trends have been observed in other countries and the latest data collection method does not restrict the numbers of drugs that CHMS participants can report. To test the hypothesis, this study aims to review the potential effects of the changes and verify the quality of reporting by the ATC drug categories before and after the changes made to the data collection method. If there are minimal effects caused by the changes in the data collection methods, the trends in the use and numbers of prescription and OTC drugs between 2007 and 2015 are also illustrated.

## Methods

The CHMS is an ongoing national survey that collects data related to health measures, biomarkers, and medication use.[14] The details of the sampling strategies and the eligibility criteria to be sampled in the CHMS could be found in Tremblay et al.[14] The eligibility criteria were similar across the four cycles, which were released before December 2017.[14] In brief, those living on reserves or in Aboriginal settlements, institutional residents, and full-time members of the Canadian Forces were not eligible for being sampled in the CHMS, less than 4% of Canadians.[14] In this study, eligible subjects were referred to as non-institutionalized Canadians. The cycles were implemented between 2007 and 2009, 2009 and 2011, 2012 and 2013, and 2014 and 2015. Based on the stratified sampling strategy, collections sites were first chosen and households were sampled from the collection sites.[14, 15] In these households, respondents were selected for interview.[14, 15] There were at least 5000 non-institutionalized Canadians sampled to draw nationally representative statistics for each cycle. The CHMS variables were cleaned according to the user guides.[16] The values representing, “not applicable”, “not stated” and “refused” were recoded to missing values. The medication-related variables in four cycles were identified, screened, summarized and merged to the household data for analysis.[17–20]

This secondary data analysis was approved by the ethics review committee at the Centre Hospitalier de l’Université de Montréal (15.115). There is no consent to participate required.

### Criteria for the assessment of the effect of the changes in medication data collection

There were three criteria assumed to be important for the assessment of the effects of the changes in medication data collection. The first criterion was the total number of the types of medications ever collected from all CHMS participants. If the limits on the maximal numbers of reported drug codes were important, it was possible that the total numbers of the medication ever reported might be less in the CHMS cycles 1 and 2 because of the censoring. Second, the trends of the numbers or the use of prescription or all drugs might be influenced by the limits. For example, the limits could restrict the total numbers of medications that could be reported and some of the medications might be skipped for the lack of reporting entries. Lastly, the trends in the proportions of diabetes, thyroid, and lipid-lowering medication may reflect their respective disease prevalence. If the trends of disease-specific medication were different from those of disease prevalence, especially when there were lower proportions of disease-specific medication use in cycles 1 and 2 compared to disease prevalence, the restrictions in data collection might be the reason.

### Medication identification

The total numbers of prescription and OTC drugs used in the past month were retrieved directly from the respective variables in four cycles (“medd100a” and related variables in cycles 1 and 2; “meufnum” in cycles 3 and 4). The numbers of prescription and all medications were labelled for individuals. For cycles 1 and 2, the ATC drug codes were searched from the variables that represented any prescription drug use, such as those beginning with “atc_1”. For OTC drugs and health products, the drug codes were retrieved from the variables beginning with “atc_2” or “atc_3”. The identified drug codes were categorized as prescription or OTC drugs. For cycles 3 and 4, the drug types, either prescription or OTC drugs, were first identified according to the drug type variable, “meuftype”. Then the ATC drug codes were extracted. The drugs codes were subsequently merged to individual data according to the individual identification numbers in the household data file. The grouping of ATC drug codes were based on the first of the five levels of the ATC classification, namely the first character of the ATC codes in Table 1. For example, the codes beginning with C represented therapeutic products targeting cardiovascular system.[21]

**Table 1:** The anatomical therapeutic chemical classification system.

	**Code**	**Contents**
**First levels of the ATC classification**		
	A	Alimentary tract and metabolism
	B	Blood and blood forming organs
	C	Cardiovascular system
	D	Dermatologicals
	G	Genito-urinary system and sex hormones
	H	Systemic hormonal preparations, excluding sex hormones and insulins
	J	Anti-infective for systemic use
	L	Antineoplastic and immunomodulating agents
	M	Musculo-skeletal system
	N	Nervous system
	P	Antiparasitic products, insecticides and repellents
	R	Respiratory system
	S	Sensory organs
	V	Various
**Disease-specific ATC classification**		
	A10	Diabetes
	H03	Thyroid medication

Note: ATC = Anatomical Therapeutic Chemical.

### Disease identification

In the CHMS survey, there were eight physician-diagnosed chronic conditions reported.[9] In Table 2, three of the chronic conditions, cardiovascular disease, diabetes, and thyroid condition, were selected for several reasons. These chronic conditions were included in the surveys through cycles 1 to 4. The questions used for interview were similar from cycles 1 to 4. The medications for the conditions were previously studied with the CHMS data.[22, 23] For each condition, there were one to eight related variables. When individuals reported “yes’ to any of the variables, they were considered to have the conditions.

**Table 2:** Identification of disease and disease-specific drug codes.

**Disease categories**	**CHMS disease reported**		**Medication**	
	Variables	CHMS concepts	Medication categories	ATC levels and codes
**Diabetes**	ccc_51	Has diabetes	Anti-diabetic	First and second levels: A10
	ccc_52a	Diabetes—insulin dependant		
	ccc_52b	Diabetes—non-insulin dependant		
	ccc_52c	Diabetes–gestational		
	ccc_53	Diabetes—age first diagnosed		
**Cardiovascular disease**	ccc_31	Has high blood pressure	Cardiovascular	First level: C
	ccc_32	Medication—high blood pressure—past month		
	ccc_34	Ever told blood cholesterol high		
	ccc_33	Ever had blood cholesterol measured		
	ccc_61	Has heart disease		
	ccc_62	Heart disease—age first diagnosed		
	ccc_63	Ever had a heart attack		
	ccc_81	Suffers from the effects of a stroke		
**Thyroid condition**	ccc_82	Has a thyroid condition	Thyroid	First and second levels: G03

ATC = Anatomical Therapeutic Chemical; CHMS = Canadian Health Measures Survey.

### Disease-specific ATC codes

At the same time, there were three types of medications separately identified for data verification in Table 1: diabetes medication,[22] thyroid drugs,[23] and cardiovascular medications. The three groups were identified mainly based on the first one or two levels of the ATC codes. The three drug types were created for the comparison with disease prevalence. If there were major discrepancies in disease prevalence and proportions of medication use especially an upward increase in medication use relative to disease prevalence observed after the changes in data collection in cycle 3, there was the possibility that some of the disease-specific ATC codes in cycles 1 and 2 might not have been properly reported partly due to the restrictions on drug reporting or the changes in medication data collection. The variables for disease identification and the corresponding drug codes were listed in Table 2.

### Trend analysis

The use and the numbers of prescription or OTC drugs were first described with mean values by cycles.[17] The drug trends were then depicted with ratios with 95% confidence intervals (CIs), compared to cycle 1 (ratio = 1). The 95% CI higher or lower than one was considered significantly higher or lower than the values in cycle 1 respectively. The trends were illustrated by the four CHMS cycles.[17] Because individuals were given weights that were designed to account for non-response and create weighted samples representative of the average population in the period of the survey, the CHMS cycles were used as the proxy for time.[17] The binary outcome of using medication or not was described in proportions. The first levels of the ATC classification codes and the three disease-specific ATC classes were also analyzed for trends.

The statistics were weighted statistics and adjusted for survey design, unless otherwise specified. The survey design required the specification of study sites, provinces, weight variables and bootstrap weights.[16] The degrees of freedom were specified as required.[16, 18, 20, 24–28] Data processing and statistical analysis were conducted with R (v3.20)[29] and RStudio (v0.98.113).[30]

## Results

The population characteristics were listed in Table 3. There were more than 29 million of non-institutionalized Canadians represented in the four cycles and about half of them were female. In the CHMS cycles 1 and 4, there were 739 and 603 types of medication ever identified from all CHMS interviewees respectively. There were 60 variables derived to summarize the use and numbers of prescription alone or prescription and OTC drugs combined. Fifty-six of them were from the first levels of the ATC classification and four were disease-specific classes in Table 1.

**Table 3:** The characteristics of the non-institutionalized canadians in the Canadian Health Measures Survey, cycles 1 to 4.

Cycles	Cycle 1	Cycle 2	Cycle 3	Cycle 4
**Weighted N with full weights**	29235444	31026646	31663898	32275596
**Number of variables**	6798	6971	9720	3066
**Study time**	2007 to 2009	2009 to 2011	2011 to 2013	2014 to 2015
**Proportions of females**	0.502	0.501	0.501	0.501
**Mean ages (years)**	39.3	38.6	39	39.3
**Mean BMI (kg/m^2^)**	26	25.8	25.9	26.2
**Mean household income (Canadian dollars)**	77818.5	80085.7	84779.2	92165.8

BMI: body mass index

### Potential impact of the changes in data collection

**Fig 1:**
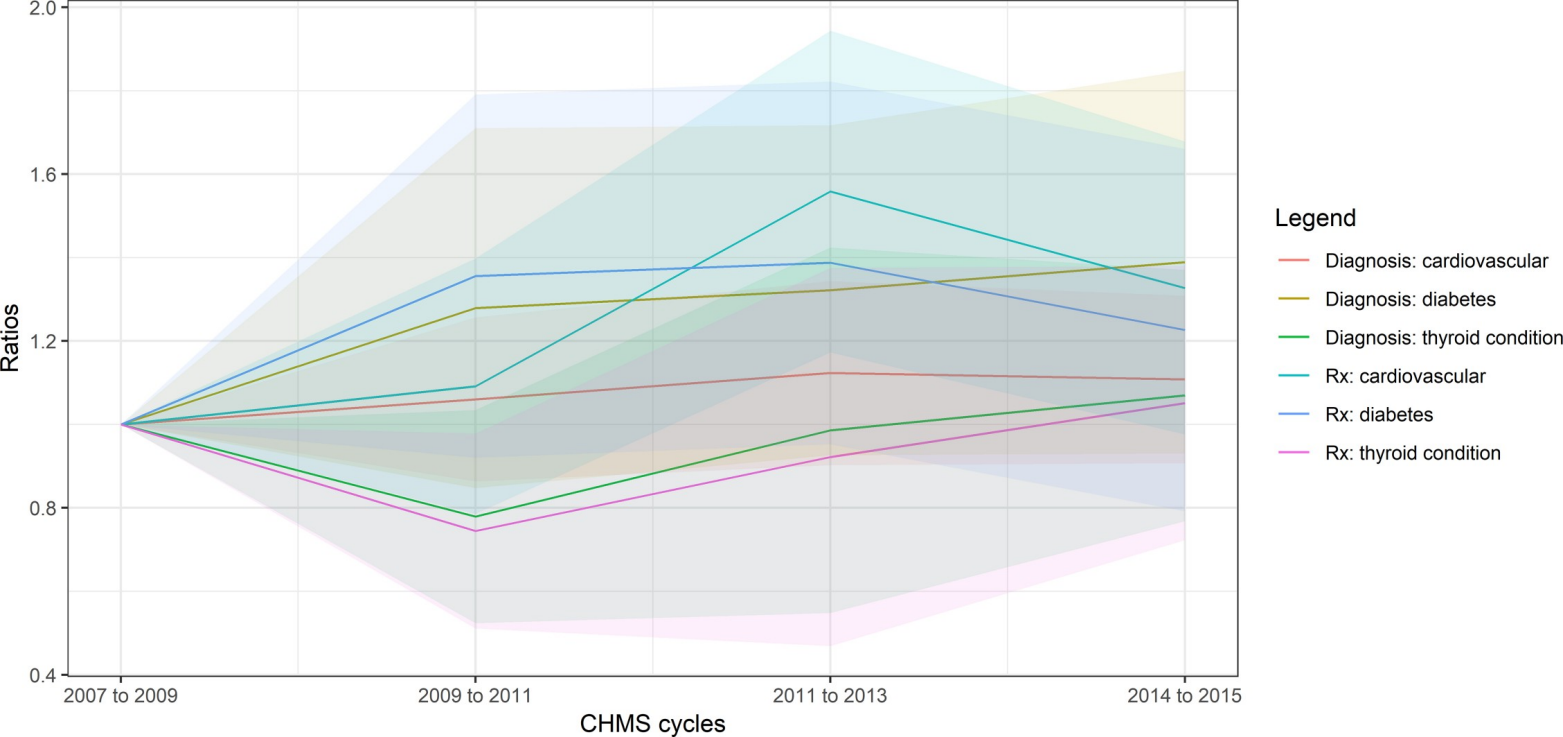
Ratios of the prevalence of cardiovascular disease, thyroid conditions and diabetes and the use of related medication. Shade area = 95% confidence intervals. Rx = drug types (see Table 1 for the drug codes).

The proportions of the use of disease-specific drugs were plotted in Fig 1, with the prevalence of cardiovascular disease, diabetes, and thyroid condition. The six curves clustered in three groups in cycles 1 and 2. The highest two curves were the diagnosis and treatment of diabetes. The two curves in the middle were the treatment and diagnosis of cardiovascular disease. The lowest two curves throughout cycles 1 to 4 represented the diagnosis and treatment for thyroid conditions. The trends of the proportions or ratios of thyroid and diabetes medication use were similar to those of the disease prevalence (see Table 1 for the drug codes and S1 Table for statistics).

In contrast, there was an abrupt increase in the use of cardiovascular drugs between cycles 2 and 3. The curve of cardiovascular drug use deviated from the prevalence of cardiovascular disease. Also, there might be a mismatch in the treatment and diagnosis for cardiovascular disease. The prevalence of cardiovascular disease remained above 57% throughout the four cycles.

There was some evidence for significant changes in disease-specific drug use and cardiovascular medication use compared to cycle 1. The proportions of using cardiovascular drugs (ATC level 1: C, ATC C in short) were below 28% throughout cycles 1 to 4. The proportion of using cardiovascular drugs (ATC C) in cycle 3 was higher than cycle 1. The proportion of using thyroid drugs did not change significantly in cycle 2 (ratio = 0.74, 95% CI = 0.51 to 0.98), compared to cycle 1.

### Trends of medication use

**Fig 2:**
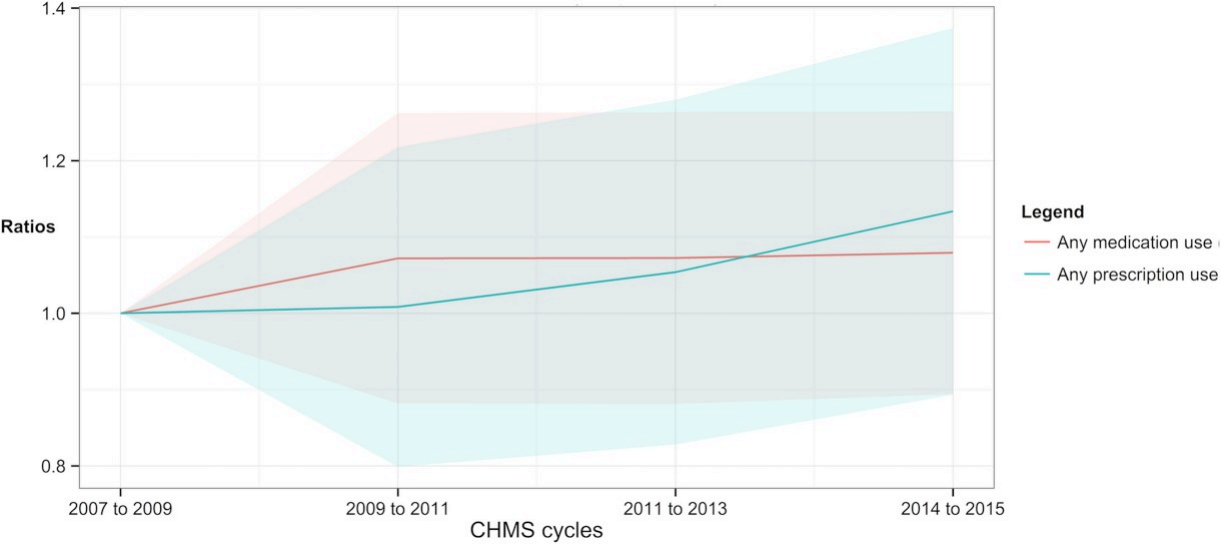
Ratios of the proportions of prescription and medication use according to the Canadian Health Measures Survey. Shade area = 95% confidence intervals.

**Fig 3:**
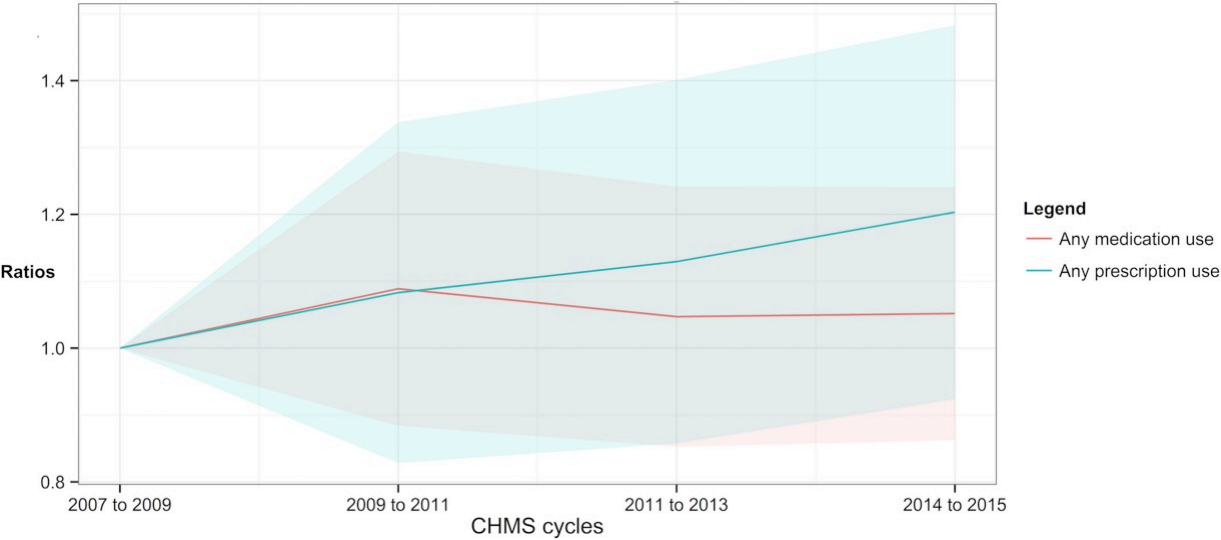
Ratios of the numbers of prescription and medication used by canadians according to the Canadian Health Measures Survey. Shade area = 95% confidence intervals.

**Fig 4:**
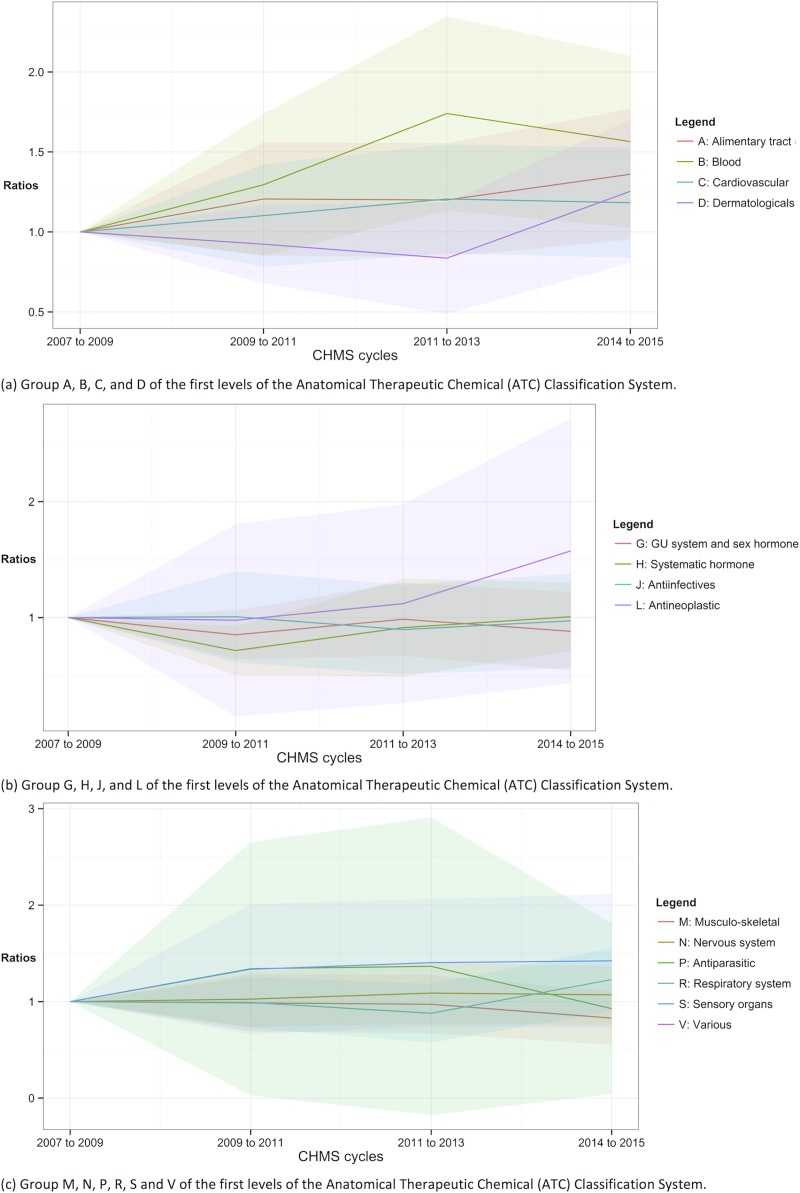
Trends of prescription use according to the ATC classification. Shade area = 95% confidence intervals.

**Fig 5:**
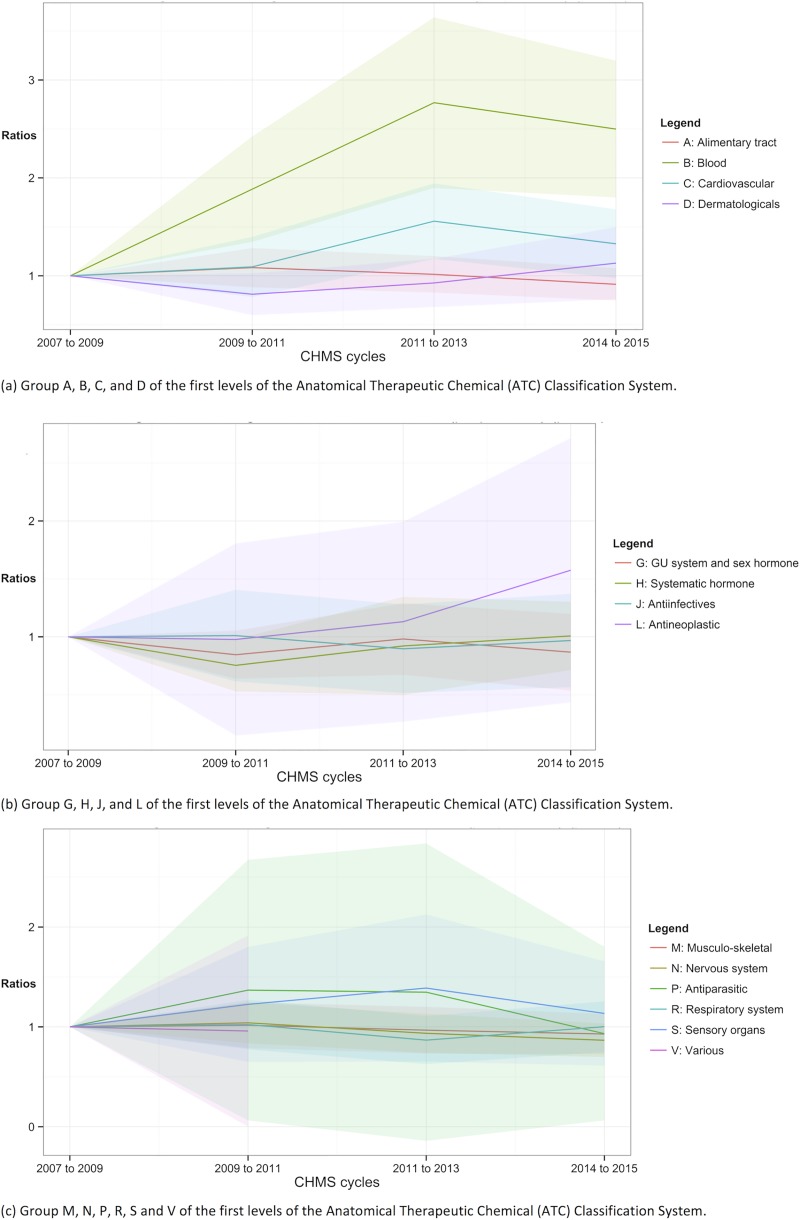
Trends of drug use according to the ATC classification. Shade area = 95% confidence intervals.

**Fig 6:**
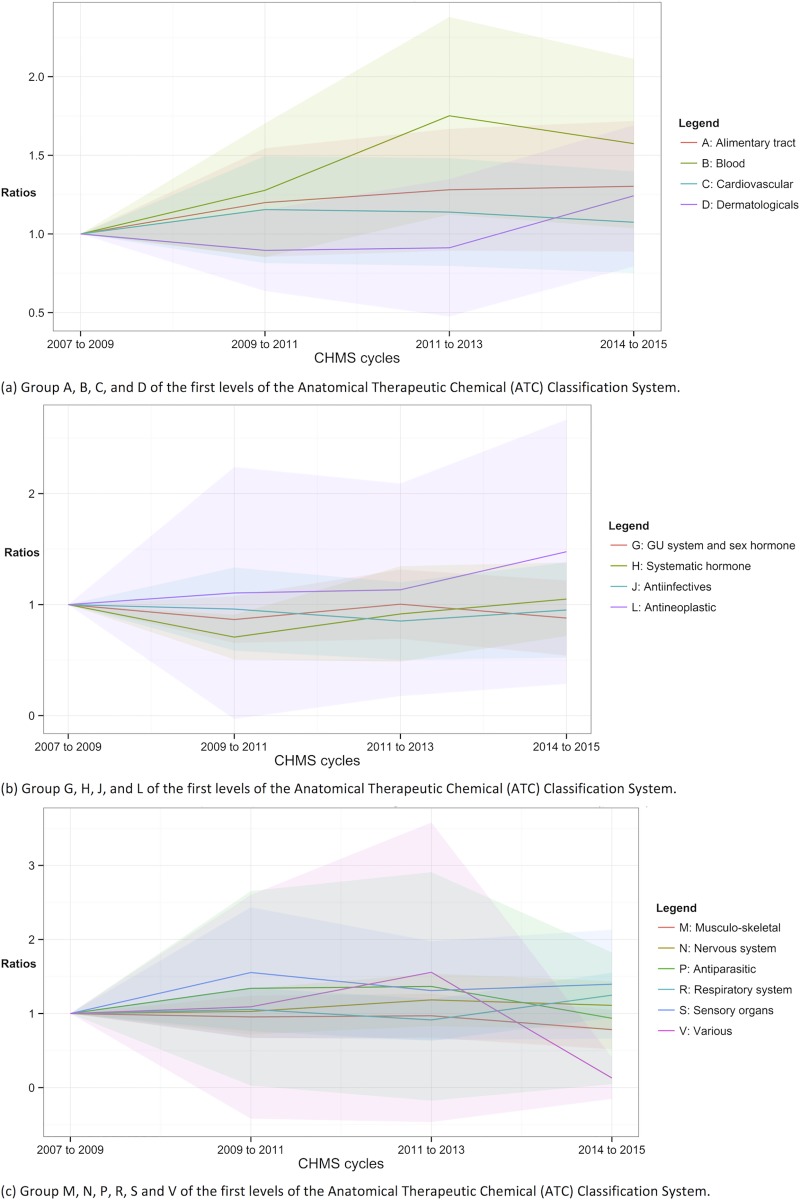
Trends of cardiovascular, diabetes, lipid-lowering and thyroid drug use. Shade area = 95% confidence intervals.

**Fig 7:**
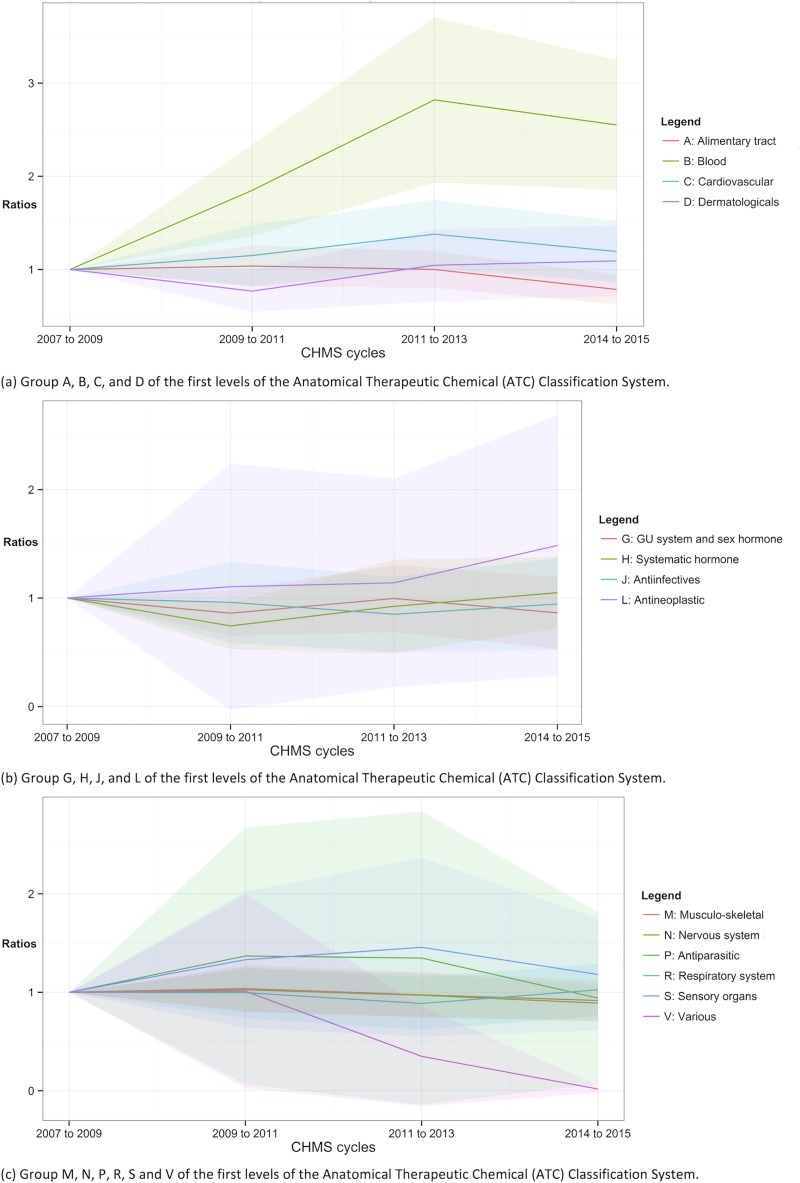
The numbers of the prescription and over-the-counter drugs from 2007 to 2015. Shade area = 95% confidence intervals.

The trends of medication use were shown in Table 4 and Figs 2 to 7. In Table 4, the proportions of using any drug, prescription and OTC, remained above 0.88 throughout the four cycles (0.90 to 0.88 from cycles 1 to 4, ratio of cycle 4 = 1.08, 95% CI = 0.89 to 1.26). The proportions of using any prescription remained more than 0.51 throughout cycles 1 to 4 (0.53 to 0.55 from cycles 1 to 4, ratio of cycle 4 = 1.13, 95% CI = 0.89 to 1.37).

**Table 4:** Trends of the proportions of the use of prescription or all drugs by the Canadian Health Measures Survey cycles.

	CHMS cycles	Ratios (cycle 1 = 1)	95% CIs		Weighted mean
**Proportions of drugs in use**	1	1.00	(1.00 to	1.00)	0.90
	2	1.07	(1.26 to	0.88)	0.90
	3	1.07	(1.26 to	0.88)	0.89
	4	1.08	(1.26 to	0.89)	0.88
**Proportions of prescription in use**	1	1.00	(1.00 to	1.00)	0.53
	2	1.01	(1.22 to	0.80)	0.51
	3	1.05	(1.28 to	0.83)	0.52
	4	1.13	(1.37 to	0.89)	0.55
**Numbers of drugs in use**	1	1.00	(1.00 to	1.00)	3.92
	2	1.09	(1.29 to	0.88)	4.07
	3	1.05	(1.24 to	0.85)	3.80
	4	1.05	(1.24 to	0.86)	3.78
**Numbers of prescription in use**	1	1.00	(1.00 to	1.00)	1.51
	2	1.08	(1.34 to	0.83)	1.59
	3	1.13	(1.40 to	0.86)	1.57
	4	1.20	(1.48 to	0.92)	1.68

Note: CHMS = Canadian Health Measures Survey; CI = confidence interval.

In Table 4 and Fig 3, the numbers of prescription medications and all drugs taken were more than 3.78 and 1.5 throughout cycles 1 to 4, respectively. The numbers of all drugs in use were from 3.9 to 3.8 between cycles 1 and 4 (ratio of cycle 4 = 1.05, 95% CI = 0.86 to 1.24). The numbers of prescription medications in use were from 1.51 to 1.68 between cycle 1 and 4 (ratio of cycle 4 = 1.20, 95% CI = 0.92 to 1.48). The numbers of prescription or all drugs was not significantly different from one for cycles 2 to 4 in Fig 3.

### Trends in medication use, by ATC classification

In Figs 4 and 5, the ratios of using any prescription and any drugs, respectively, were plotted according to the first-level categories of the ATC classification. The ratios and the proportions according to the first-level categories could be found in S2 and S3 Tables. The drugs of the “various” category (ATC V) could not be estimated due to insufficient sample sizes for prescription use in cycles 1 to 4 in Fig 4 and for the use of both prescription and OTC drugs in cycles 3 and 4 in Fig 5. The proportions of using prescription for blood and blood forming organs (ATC B) use were significantly higher in cycles 3 and 4, suggesting increasing trends [ratio of cycles 3 and 4 = 1.740 (95% CI = 1.13 to 2.35) and 1.56 (95% CI = 1.03 to 2.10) respectively]. Hormonal prescription use, excluding sex hormone and insulins, (ATC H) in cycle 2 was significantly less prevalent (ratio = 0.72, 95% CI = 0.51 to 0.93). The proportions of using other prescription medications in the following categories throughout cycles 2 to 4 were not significantly different from those in cycle 1 in Fig 4: ATC A, C, D, G, J, L, M, N, P, R, and S.

In Fig 5, the trends of using any drugs, including prescription and OTC drugs were shown. The use of the drug for blood and blood forming organs (ATC B) became significantly more prevalent from cycles 2 to 4 (ratio = 1.89, 2.77, and 2.50 respectively). The use of cardiovascular drugs (ATC C) in cycle 3 was significantly higher than cycle 1 (ratio of cycle 3 = 1.56, 95% CI = 1.17 to 1.94). The prevalence of use of hormonal drugs (ATC H) in cycle 2 was lower (ratio of cycle 2 = 0.75, 95% CI = 0.53 to 0.98). The use of any drugs of the other categories between cycles 2 and 4 was not statistically significantly different from that in cycle 1: ATC A, D, G, H, J, L, M, N, P, R, S, and V.

### Trends in the number of medications, by ATC classification

Figs 6 and 7 show the ratios of the number of prescription or all drugs, respectively, throughout cycles 1 to 4. The detailed statistics were in S4 and S5 Tables. The numbers of prescription for blood and blood forming organs (ATC B) were higher in cycles 3 and 4 in Fig 6 [ratio = 1.75 (95% CI = 1.12 to 2.38) and 1.57 (95% CI = 1.04 to 2.11) respectively]. The numbers of hormonal prescription (ATC H) were lower in cycle 2 compared to cycle 1 (ratio of cycle 2 = 0.71, 95% CI = 0.50 to 0.91). The change in the number of the prescription medications of the other categories in use between cycles 2 and 4 was not statistically significant from cycle 1: ATC A, C, D, G, J, L, M, N, P, R, S, and V.

There were significantly more prescription or OTC drugs for blood and blood forming organs (ATC B) in use in cycles 2 to 4 than cycle 1 (ratio = 1.85, 2.82, and 2.55 respectively). There were significantly more cardiovascular drugs (ATC C) in use in cycle 3 than cycle 1 (ratio of cycle 3 = 1.38, 95% CI = 1.01 to 1.75). There were significant differences in the numbers of “various” drugs (ATC V) in use in cycle 3 and 4 [ratio = 0.35 (95% CI = -0.16 to 0.85) and 0.02 (95% CI = -0.02 to 0.06) respectively]. The numbers of the drugs of the other categories were not significantly different from cycle 1: ATC G, J, L, M, N, P, R, and S.

## Discussion

To the authors’ knowledge, the restrictions on drug reporting in the CHMS cycle 1 and 2 have never been studied before, and are fundamental to the studies that aim to use the CHMS data to understand the patterns of medication use in Canada. After confirming that the changes to medication data collection after the CHMS cycle 2 may not have important or significant impact on data quality, we demonstrate the results of trend analysis of prescription or OTC drug use.

There are several findings according to the trends of medication use. The first one is about the potential impact of data collection changes on the trends. The others are about the trends that we discovered based on population representative data. First, the changes made to the method of drug information collection may have minimal effects on the drug reporting, i.e. little censoring of drug codes due to the limits on the numbers of drugs reported in the CHMS cycles 1 and 2. This is related to the criteria we have established for this research objective. The numbers of all types of medication according to the ATC codes reported by all interviewees have been decreasing in contrast to the increasing trend in the use of certain prescription medications. This opposes the fact that more drugs have been approved and are now used in the market.[31] This may suggest that the diversity of drug use is in decline. Marketing, culture, and other factors may be involved in this decline.[32] The exact causes to this finding may need to be investigated in the future. There is a lack of prevalent upward bending in the medication use or numbers of medications between CHMS cycles 2 and 3 to support the hypothesis that changes in medication data collection lead to censoring of information in the CHMS cycles 1 and 2, except for cardiovascular drug use that abruptly increased after cycle 2.

Second, only prescription medications or all drugs of one ATC level-one classification, blood and blood forming agents, have been more frequently used in cycles 2 to 4, compared to cycle 1. The use of cardiovascular drugs, prescription only or all, has been more prevalent in cycle 3, implemented between 2011 and 2013. A significant decline in the use of hormonal prescription medications, excluding sex hormones and insulin, was only observed in cycle 2. These findings are similar to the significant changes in prescription drug use in the US between 1999 and 2012, where increases in cardiovascular or anticoagulant prescription use and decrease in hormonal products have been observed.[7] These trends may be related to the fluctuations in disease prevalence and evolving medical practices. In this study, we find that disease prevalence is an important factor. Throughout four CHMS cycles, the use of thyroid and diabetes drugs corresponds well with the disease prevalence among the CHMS participants. In the CHCMS cycles 1 and 2, cardiovascular drug use aligns with disease prevalence well. Then the changes in clinical guidelines may contribute to the rapid increase in cardiovascular drug use, such as the emphasis on the preventive role of aspirin and lipid-lowering agents.[33] The role of aspirin (ATC code: B01AC06) has been stressed since 2009 for the primary and secondary prevention of cardiovascular events.[34, 35] Lipid-lowering agents, statin (ATC codes mostly beginning with C10), have been recommended for patients with type 2 diabetes regardless of their lipid profile since 2008.[36, 37] The changes in medical guidelines may be one of the reasons why cardiovascular drugs have been used more frequently than disease prevalence. The decrease in sex hormones in the CHMS is similar to the trend in the US that have been linked to the decrease in non-contraceptive hormone use, particularly conjugated estrogens, after the release of the Women’s’ Health Initiative Hormone Therapy Trial.[7]

Third, there are differences between disease prevalence and proportion of drug use, especially for cardiovascular conditions and related medication. There are factors discussed and tested in the literature, such as inappropriate provider-patient communication[38] and poor disease awareness[39, 40]. It requires further investigation to understand the associations with these factors.

The last finding is that we did not find significant increases in the overall trends in prescription or OTC medications in Canada based on a nationally representative survey from 2009 to 2015. Though increasing trends of medication use have been observed in other countries.[1, 2]

To our knowledge, this is the first study to describe the overall trends of prescription and OTC medication use in Canada. The findings in our study show that it feasible to conduct trend analysis with the medication data in the CHMS. Several significant trends in drug use have been identified. Some of them can be partly explained by known factors, such as the changes in the clinical guidelines for cardiovascular drugs and the decrease in sex hormone use due to newly published trial results.[7] We encourage researchers to be actively engaged with national data, such as the CHMS, to understand the underlying causes of these trends. In the future, there are still issues to be investigated, such as trends in specific drugs, drug use in certain population groups, and the exact causes of more intense use of cardiovascular drugs.

### Strengths and limitations

This study has several strengths including national representativeness, systematic data collection, small proportions of missing information in drug codes, and comparison with disease prevalence. However, there are several limitations to this study. First, this is an analysis of repeated cross-sectional surveys focusing on non-institutionalized Canadians and those living outside reserves[16, 41]. It is likely that medication for injury and acute or severe conditions is not included for study. The drugs being used by those above the age 79 years were not surveyed and the results from the CHMS might underestimate the numbers of drugs in use.[9] It requires caution to interpret the results and is necessary to understand the population under study. Second, the impact of the changes in the data collection methods may require further review. This study is an initial attempt to do so. Third, there are limited sample sizes in certain drug categories, especially the “various” drug category (ATC V) that may be subject to the changes in coding practice in health care (personal communication with Statistics Canada). Fourth, the age range of the CHMS participants is wide, from three to 79 years. Our analysis may not be sensitive to the drug trends that exist only in certain subpopulation, such as adolescents or the elderly. Fifth, the observation time might not be long enough to identify significant increase or decrease in drug use in most of the drug classes in this nationally representative survey, compared to the 13-year follow-up in the US.[7] Lastly, the doses of the drugs are not documented. It may not be feasible to identify medications not distributed by pharmacies or clinics among the CHMS participants. Without information on doses and drugs that may not be obtained from health professionals or pharmacies, it may be difficult to assess the magnitude of ongoing opioid epidemic[42, 43] based on the CHMS data.

## Conclusions

There is an increasing trend in the use of blood and blood forming agents through cycles 2 to 4 and cardiovascular drugs in cycle 3. There have been restrictions on the drug reporting in the CHMS cycles 1 and 2. The removal of the limits on medication data collection might not have an important impact on the reporting of the use of prescription or OTC drugs based on several findings. First, the numbers of the types of medications identified from all participants decreased between cycle 1 and 4. Second, the rapid increase of the trends of medication use is not prevalent in 14 groups of medications stratified by the first level of the ATC classification between cycles 2 and 3, except for cardiovascular drugs. Lastly, the proportions of thyroid and diabetes medication use increase proportionally with disease prevalence between cycles 1 and 4. The impact of the limits on the numbers of medication that can be reported in the CHMS cycles 1 and 2 may be minimal. Trend analysis of medication use with the CHMS cycle 1 to 4 data is feasible. Trends of specific drugs in subpopulations and the associations with disease prevalence and prescribing practices may need to be further investigated.

## Supporting information

S1 Table Prevalence of cardiovascular conditions, thyroid conditions and diabetes and the proportions of using related drugs. (XLSX)

S2 Table Proportions of prescription use based on the first levels of ATC classification. (XLSX)

S3 Table Proportions of prescription and over-the-counter drug use based on the first levels of ATC classification. (XLSX)

S4 Table Numbers of prescription use based on the first levels of ATC classification. (XLSX)

S5 Table Numbers of prescription and over-the-counter drug use based on the first levels of ATC classification. (XLSX)

## Abbreviations

AHFS: American Hospital Formulary Service
ATC: Anatomical Therapeutic Chemical
BMI: body mass index
CHMS: Canadian Health Measures Survey
OTC: over-the-counter
